# Rare pulmonary embolism in a pregnant patient: A primary diffused pulmonary artery myxofibrosarcoma case report

**DOI:** 10.3389/fonc.2022.956236

**Published:** 2022-08-25

**Authors:** Kanghui Xiang, Lu Liu, Hui-Jun Li, Guang-Wei Zhang

**Affiliations:** ^1^ Department of Medical Oncology, The First Hospital of China Medical University, Shenyang, China; ^2^ Key Laboratory of Anticancer Drugs and Biotherapy of Liaoning Province, The First Hospital of China Medical University, Shenyang, China; ^3^ Liaoning Province Clinical Research Center for Cancer, The First Hospital of China Medical University, Shenyang, China; ^4^ Key Laboratory of Precision Diagnosis and Treatment of Gastrointestinal Tumors, Ministry of Education, The First Hospital of China Medical University, Shenyang, China; ^5^ Department of Cardiac Surgery, The First Hospital of China Medical University, Shenyang, China; ^6^ Shenyang Medical and Film Science and Technology Co. Ltd., Shenyang, China; ^7^ Smart Hospital Management Department, The First Hospital of China Medical University, Shenyang, China

**Keywords:** pulmonary embolism, low-grade myxofibrosarcoma, pregnancy, operation, ECMO - extracorporeal membrane oxygenation

## Abstract

A 37-year-old female patient presented with shortness of breath, cough, and chest pain complaints from the 12th week of her first pregnancy. At the 28th week, labor induction had to be performed because of severe dyspnea and hyoxemia. Thereafter, a diffused pulmonary embolism was detected by echocardiography and CT angiography, without histological diagnosis. Pulmonary endarterectomy was performed, and it was found during operation that a huge, lobular mass originated in the posterior wall and extended throughout the vasculature of both lungs, and a mucinous pellicle covered the entire pulmonary endothelium. Pathology revealed a low-grade myxofibrosarcoma with positive vimentin and SMA, partially positive CD-34.

## Introduction

Low-grade myxofibrosarcoma (LGMFS) is a low-grade malignant mesenchymal tumor, which usually arises in the extremities of 60–80-year-old patients and rarely originates in the pulmonary artery of young patients. Thereby, it is often misdiagnosed as chronic pulmonary thromboembolism. Furthermore, surgical operation for these patients is still a challenge. Here, we report a rather rare case with incidence of LGMFS during pregnancy.

## Case report

A 37-year-old female patient presented with shortness of breath, cough, and chest pain complaints from the 12th week of her first pregnancy. However, no special examination or treatment was performed. Thereafter, these symptoms were gradually worsened. At the 28th week of pregnancy, labor induction had to be performed because of severe dyspnea and hyoxemia. Two weeks later, she was admitted to our hospital for continually aggravated symptoms. She reported an apparent distress at rest but no syncope, palpitations, lower-extremity edema, weight loss, fever, rashes, or arthritis. There was a history of hysteromyoma defined by transvaginal ultrasound but no history of tobacco, estrogen, appetite suppressant, or illicit drug use, deep-venous thrombosis, or familial pulmonary hypertension or thromboembolic disease. No positive sign was found in physical examination.

**Figure 1:**
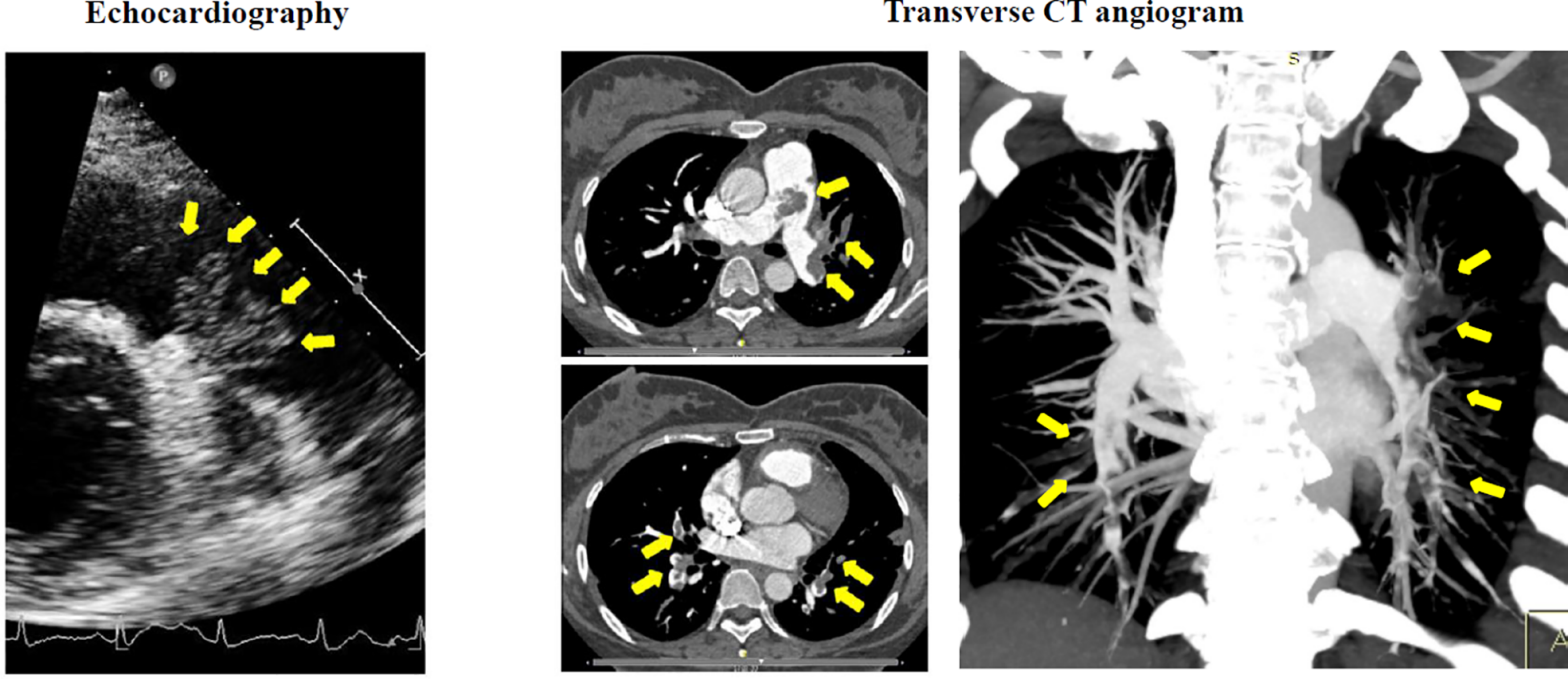
Preoperative echocardiography demonstrated a large lobulated mass (arrows) attached by a pedicle to the posterior wall of pulmonary truck. Transverse CT angiogram revealed a diffused occlusion of both pulmonary arteries (arrows).

Chest radiography showed patchy alveolar opacities in both lower lobes, right-ventricular enlargement, and prominent central pulmonary artery. Echocardiography revealed a lobulated mass occupying attached by a pedicle to the posterior wall of the main pulmonary artery, a right-ventricular enlargement with an estimated pulmonary artery systolic pressure of 79 mmHg and normal left ventricular size and function. Subsequently, diffuse pulmonary embolism was detected by CT angiography (**Figure 1**). Electrocardiography (ECG) evidenced Wolff–Parkinson–White syndrome. Lower-extremity duplex ultrasonography was negative. Laboratory analysis results showed white blood cell count (WBC) 13.66 KU/l, erythrocyte sedimentation rate (ESR) 66 mm/h, C-reactive protein (CRP) 86.3 mg/l, and D-dimer 1,378 μg/l. Moreover, cancer antigen (CA)-125, CA-15-3, CA-19-9, and carcinoembryonic antigen (CEA) results were normal.

**Figure 2:**
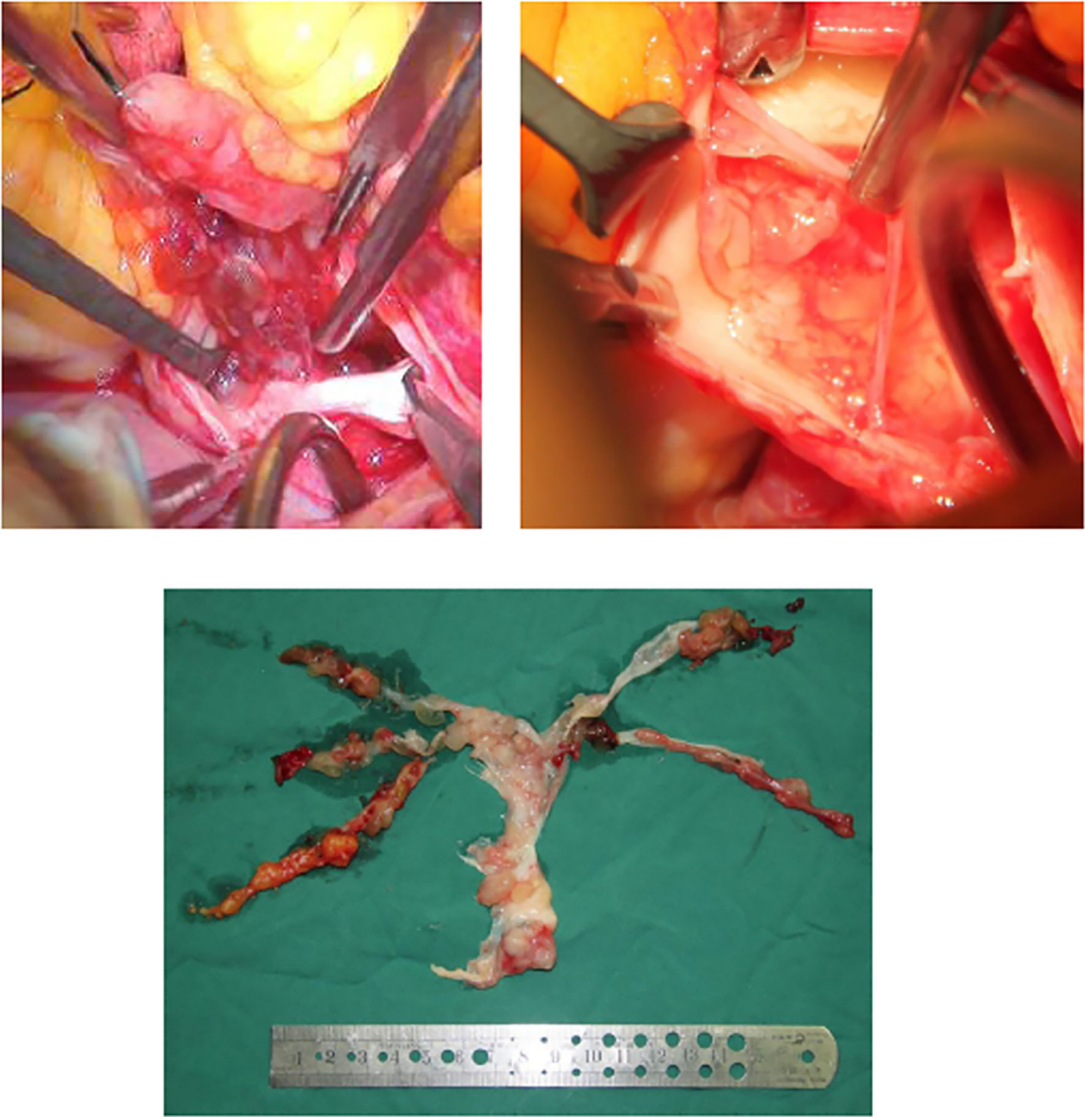
It is proved during operation that a huge, lobular mass originated in the pulmonary trunk, diffuse growth into both pulmonary arteries was observed, and the entire pulmonary endothelium was covered by a thin mucinous pellicle.

**Figure 3:**
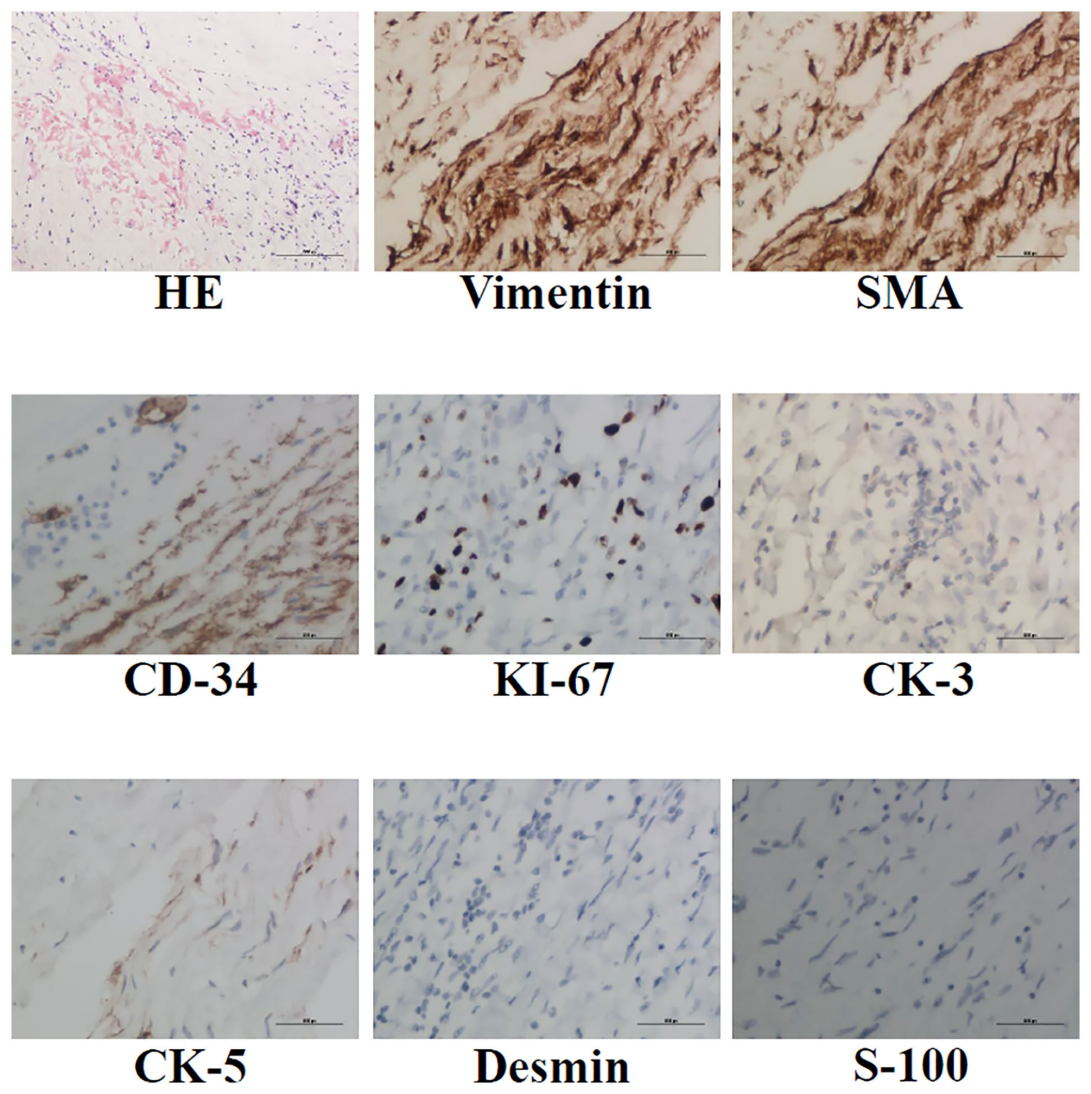
HE, anti-vimentin, SMA, CD-34, CK-3, CK-5, desmin and s-100 immunohistochemistry staining was performed to identify LGMFS.

**Figure 4:**
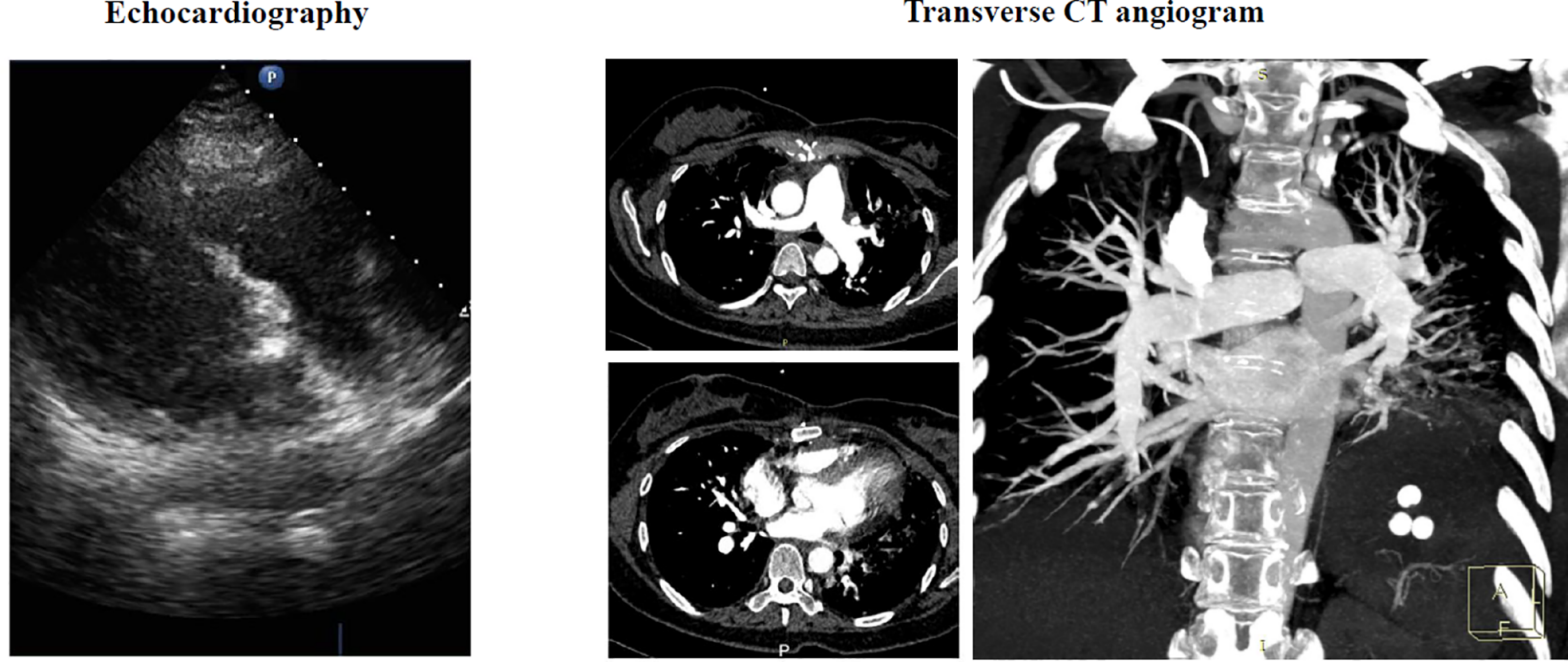
Postoperative echocardiography and transverse CT angiogram showed that most tumor tissue of both lungs has been resected with a little residual occlusion.

The operation was performed at undiagnosed conditions, due to severe dyspnea and hyoxemia. Femoral cannulation of cardiopulmonary bypass (CPB) was performed for preparation of possible ECMO. When the pulmonary trunk was incised, it was found that a huge, lobular mass originated in the posterior wall and extended throughout the vasculature of both lungs, and a mucinous pellicle covered the entire pulmonary endothelium (**Figure 2**). Because the tumor has invaded into subsegmental and distal arteries, it was impossible for a complete resection. Deep hypothermic circulatory arrest (DHCA) was used for better exposure to resect more tumor tissue in distal pulmonary arteries. The patient suffered from a severe pneumorrhagia, immediately following aortic cross-clamp opening. Therefore, it was difficult for withdrawal of CPB and the patient had to undergo ECMO support. Seven and 10 days after operation, ECMO and tracheal intubation were removed, respectively. Pathology revealed a LGMFS with positive vimentin and SMA, partially positive CD-34, and negative CK-3, CK-5, desmin, and s-100. HE staining showed that spindle cells proliferated and were crowded, with myxoid stroma around them. The percentage of Ki-67-positive cells was about 30% (**Figure 3**). A subtotal resection was confirmed by postoperative CT angiography and echocardiography (**Figure 4**). Twenty-two days postoperatively, the patient was discharged with a good recovery, followed by chemotherapy (pazopanib) and lung radiotherapy. Two years after operation, the patient reported that she had no obvious respiratory symptoms, but unfortunately refused CT or echocardiography examination, and thereafter was lost to follow-up due to poor compliance.

## Discussion

Pulmonary thromboembolism is a common complication after pregnancy (1). The patient had similar medical histories and significantly increased D-dimer, which supported that diagnosis. However, echocardiography found a lobulated mass occupying in the pulmonary trunk, increasing three probabilities of diagnosis, namely, benign myxoma, malignant sarcoma, and myxoma combined with thromboembolism. In addition, uterine leiomyosarcoma cannot be also excluded, due to a past history of hysteromyoma. In CT scan views, however, there is no a significant wall eclipsing sign as described by Gan et al. (2), no contrast enhancement of the mass and extravascular spread of the lesion as described by Scheffel et al. (3), or no other characteristic signs of malignant sarcoma. Therefore, the diagnosis was rather confused before operation.

Because of the similar clinical presentations and no specific biomarker, it is very difficult to differentiate pulmonary artery tumor and thromboembolism without histological diagnosis, which is often impossible preoperatively (2), so that most pulmonary artery tumor patients were misdiagnosed with thromboembolism before surgical intervention, consequently receiving inappropriate therapeutic strategies such as long-term anticoagulant treatment (2, 3). Therefore, regardless of whether it is benign or malignant, it is suggested that the patients with a severe pulmonary embolism should undergo operation (4, 5). This is because the median survival of time of untreated patients after diagnosis of malignant tumor is only several months (range 1.5–5.5 months) (6, 7), which can be significantly increased by surgical treatment, even to 5–10 years reported by Tavora et al. (8).

Due to better prognosis in patients with complete resections than incomplete resections (8), it is advocated that DHCA should be used to for a clear exposure for a more complete resection of LGMFS in distal pulmonary arteries. The pulmonary artery hypertension from chronic embolism and endothelial injury from surgical procedure severely damages heart and lung function, which usually needs ECMO support (9). Thus, femoral cannulation is a reasonable choice, fulfilling a direct conversion from CPB to ECMO without re-cannulating. It is suggested that chemotherapy and lung radiotherapy were performed after surgical resection to prevent recurrence, although the role of these methods is still controversial and the outcome is uncertain (10, 11).

It has been reported that vimentin and SMA are used for identification of myxofibrosarcoma. In this patient, besides the tumor cells being vimentin and SMA positive, these cells partially expressed CD-34, perhaps representing a novel myxofibrosarcoma cell line or suggesting a conversion from myxofibrosarcoma cells to endothelial cells. Similar reports are absent.

## Data availability statement

The raw data supporting the conclusions of this article will be made available by the authors, without undue reservation.

## Ethics statement

Written informed consent was obtained from the individual(s) for the publication of any potentially identifiable images or data included in this article.

## Author contributions

KX carried out data collection and immunohistochemical analysis. LL and G-WZ participated in clinical treatment. H-JL and G-WZ drafted the manuscript. All authors read and approved the final manuscript.

## Conflict of interest

Author H-JL was employed by Shenyang Medical and Film Science and Technology Co. Ltd., Shenyang, China.

The remaining authors declare that the research was conducted in the absence of any commercial or financial relationships that could be construed as a potential conflict of interest.

## Publisher’s note

All claims expressed in this article are solely those of the authors and do not necessarily represent those of their affiliated organizations, or those of the publisher, the editors and the reviewers. Any product that may be evaluated in this article, or claim that may be made by its manufacturer, is not guaranteed or endorsed by the publisher.
